# Associations Between Upright Cardiovascular-Autonomic Characteristics, Temporal Gait Measures, and Mediolateral Plantar Center-of-Force Measures in Adults with Developmental Disabilities: An Exploratory Cross-Sectional Study

**DOI:** 10.3390/medicina62101968

**Published:** 2026-10-12

**Authors:** Ji-Hyun Nam, Han Suk Lee

**Affiliations:** Department of Physical Therapy, Eulji University, Seongnam 13135, Republic of Korea

**Keywords:** developmental disabilities, heart rate variability, gait variability, gait symmetry, autonomic nervous system

## Abstract

*Background and Objectives*: Adults with developmental disabilities may show altered gait and autonomic regulation, but their relationship during upright function remains unclear. This study examined associations between upright cardiovascular-autonomic characteristics, temporal gait measures, and mediolateral plantar center-of-force (COF) measures and secondarily compared intellectual disability (ID) and autism spectrum disorder (ASD) groups. *Materials and Methods*: This cross-sectional study included 24 adults. Measures were the change in heart rate from supine to standing (ΔHR), standing root mean square of successive differences (RMSSD), standard deviation of normal-to-normal intervals (SDNN), and normalized high-frequency power [HF (nu)]. F-Scan 3-Box outputs from three 6-meter walks provided eight gait measures, including trial-to-trial coefficients of variation (CV) and symmetry index (SI) of temporal variables. Thirty-two primary partial rank correlations were adjusted for age, sex, and body mass index (BMI), with Benjamini–Hochberg false discovery rate (BH-FDR) correction. *Results*: Standing HF (nu) and stride time SI showed a nominally significant negative association (partial ρ = −0.663, unadjusted *p* = 0.0071; 95% CI [−0.877, −0.227]), but this association did not remain significant after BH-FDR correction (q = 0.228). No primary association survived FDR correction. Additional adjustment for diagnosis yielded ρ = −0.672 (unadjusted *p* = 0.0085). Standing RMSSD and stance time CV were not significantly associated (ρ = −0.527, *p* = 0.053). No primary gait measure differed significantly between ASD and ID. *Conclusions*: The observed standing HF (nu)–stride time SI association is exploratory and did not survive correction for multiple testing. The findings do not establish stronger temporal than spatial associations, clinical benefit, or a causal mechanism. Larger studies with detailed heart rate variability (HRV) and gait-event measurements are needed.

## 1. Introduction

Developmental disabilities comprise a broad group of conditions affecting the developing nervous system that may involve impairment in cognition, motor, language, behavioral and social skills [1]. Intellectual disability (ID) and autism spectrum disorder (ASD) are neurodevelopmental conditions included within this broad clinical context [2]. These developmental difficulties begin in childhood and can persist into adulthood, affecting daily functioning and social participation [1,2]. Accordingly, functional problems in adults with developmental disabilities should be understood not only in terms of reduced task performance, but also in terms of whether individuals can conduct their daily lives and participate in meaningful activities. Among adults with ID, participation in everyday, social, and leisure activities has been identified as an important component of quality of life [3], while limitations in community inclusion and living skills may persist into adulthood in ASD [4].

Walking supports daily activity and community participation. Adults with ID have a high prevalence of falls [5], and adults with ASD show differences in gait speed, double-stance time, and trunk-to-head stabilization [6]. Locomotion involves spinal central pattern generators (CPGs), supraspinal regulation, and brainstem postural pathways [7,8]. These mechanisms provide physiological background, but the present study measured temporal gait summaries and plantar center-of-force (COF) measures. Greater gait variability and a wider base of support have been reported in ID [9], with additional temporal changes during dual-task walking [10]. COF deviation and excursion describe plantar force trajectories [11], complementing temporal measures without directly establishing functional stability or fall risk.

Locomotor–respiratory coupling [12], vestibulo-autonomic pathways [13], insular integration [14], and cardiolocomotor synchronization during walking or running [15] offer a rationale for studying autonomic and gait characteristics together. However, these mechanisms were not measured here. The present correlations cannot establish physiological coupling, rhythm generation, or a specific neural pathway.

Stride and stance times describe the timing of repeated gait events. Their trial-level means, between-trial coefficients of variation (CVs), and bilateral symmetry indices (SIs) characterize different aspects of temporal organization. Stride time SI was included as a complementary descriptor of bilateral temporal gait organization to explore whether upright cardiovascular–autonomic characteristics were associated with left–right differences in trial-level stride time means. It does not represent cycle-matched asymmetry or directly measure functional stability.

Differences in autonomic regulation have also been reported separately in adults with ID and ASD. Adults with ID demonstrated blunted heart rate, hemodynamic, and heart rate variability (HRV) responses to active standing and head-up tilt, including reduced parasympathetic modulation in response to orthostasis [16]. Adults with ASD have also shown lower resting high-frequency (HF) power and root mean square of successive differences (RMSSD) than comparison participants, consistent with relatively reduced parasympathetic modulation [17]. Although spatiotemporal gait alterations and autonomic dysregulation have each been described in adults with ID and ASD, these domains have largely been examined separately. Few studies have directly examined how autonomic regulation during upright standing relates to temporal gait measures and plantar COF measures within the same individuals.

Accordingly, this exploratory cross-sectional study investigated associations between upright cardiovascular-autonomic characteristics, temporal gait measures, and plantar COF measures in adults with ID or ASD. ΔHR characterized the change in heart rate from supine to standing, while standing RMSSD, the standard deviation of normal-to-normal intervals (SDNN), and HF in normalized units [HF (nu)] characterized the recorded upright autonomic state. COF deviation and excursion quantified selected features of the mediolateral plantar center-of-force trajectory; the mean, coefficient of variation (CV), and symmetry index (SI) of stride and stance time described temporal gait characteristics. Associations were adjusted for age, sex, and body mass index (BMI); diagnostic-group comparisons were secondary.

No individual association was prespecified as the principal hypothesis; all 32 variable pairs were examined on an equal exploratory basis. Differences in nominal significance between domains were not interpreted as evidence of differences in association strength.

## 2. Materials and Methods

### 2.1. Participants

This cross-sectional observational study included community-dwelling adults recruited from a lifelong education center for developmental disability in G City, Republic of Korea. Eligibility required age ≥18 years, a physician-confirmed diagnosis of developmental disability, specifically ID or ASD, documented in the center’s records at enrollment, the ability to walk, and cooperation with testing. None of the enrolled participants had coexisting ASD and ID diagnoses; no allocation rule for participants with both diagnoses was required. Exclusion criteria included uncontrolled frequent or severe seizures within the previous 3 months, acute cardiopulmonary or orthopedic disease, and recent surgery or fracture. The investigators did not independently re-evaluate the recorded diagnoses. Written consent was obtained from parents or legal representatives, and willingness to participate was confirmed with each participant. The study was approved by the E University Institutional Review Board (EU25-077, 13 February 2026).

### 2.2. Recruitment and Assessment

**Figure 1:**
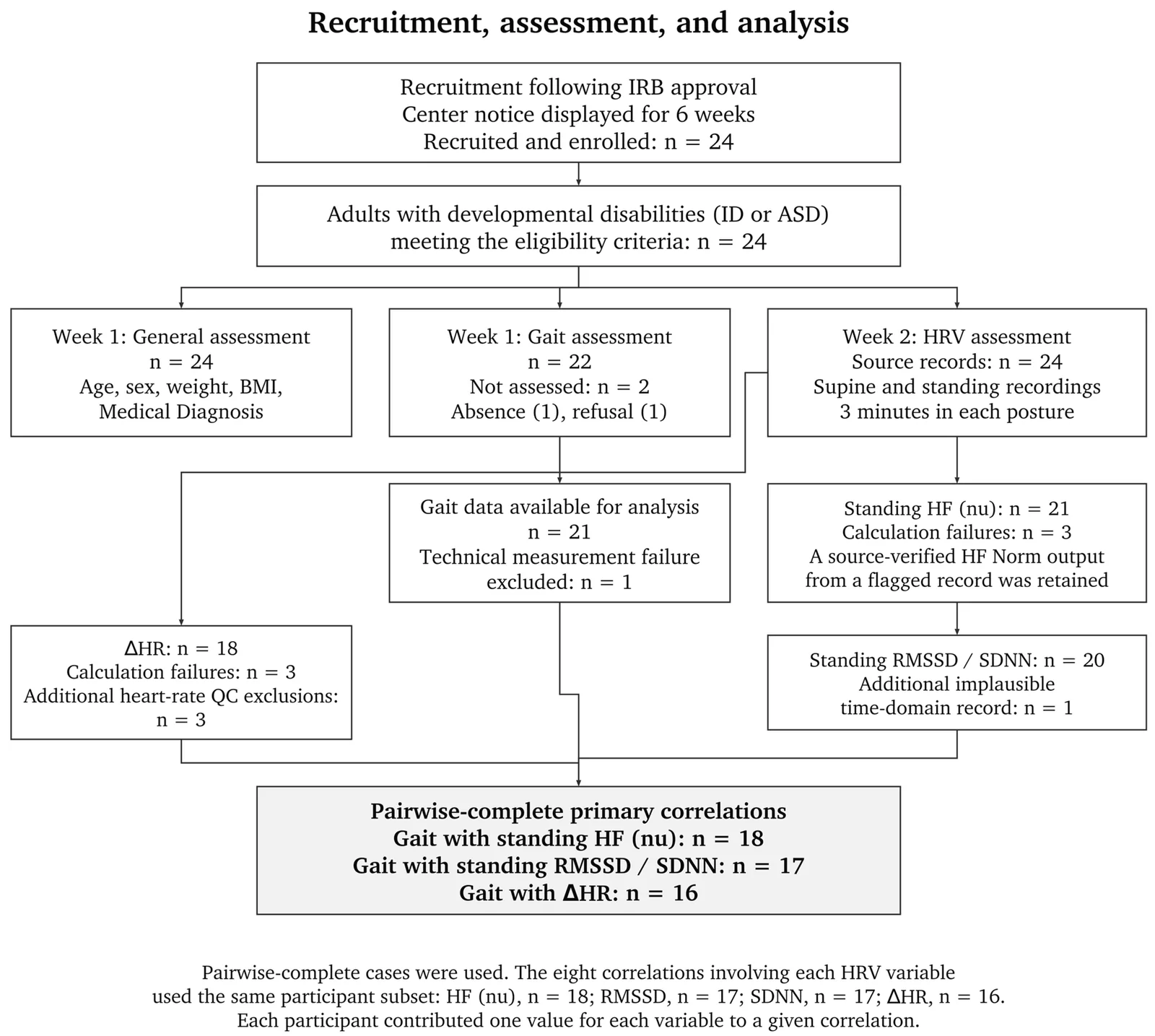
Recruitment, assessment sequence, and variable-specific data availability. In week 1, general records were available for 24 participants and gait testing was performed in 22; one unusable measurement left 21 gait datasets. HRV input records numbered 24. Valid HRV counts before pairing were 21, 20, and 18 for HF (nu), RMSSD/SDNN, and ΔHR, respectively; paired correlation counts were 18, 17, and 16. Gait-test noncompletion comprised absence (*n* = 1) and refusal (*n* = 1); one completed assessment was excluded because of a technical measurement failure.

Following IRB approval, the center displayed a recruitment notice for six weeks to adults with developmental disabilities enrolled in its comprehensive program. Parents or legal representatives of all 24 eligible adults had access to the notice, and all 24 participants were subsequently enrolled in the study. Gait assessments began on 30 March 2026. During the first assessment, participants’ general characteristics were assessed, and gait testing was performed. HRV assessment followed in the second week. One physical therapist and two trained research assistants conducted assessments at the same site on scheduled days between 10:00 and 11:30. Figure 1 summarizes the assessment sequence and variable-specific data availability.

### 2.3. Cardiovascular-Autonomic Assessment

Cardiovascular-autonomic characteristics were assessed using the SA-3000P system (Medicore Co., Ltd., Seoul, Republic of Korea). HRV parameters were analyzed using the HRV(DDR) software (version 5.0.0; Medicore Co., Ltd., Seoul, Republic of Korea). Cardiac signals were acquired using the ECG-based acquisition method of the SA-3000P system. HRV testing took place in the week following gait assessment, within the same 10:00–11:30 morning window. Participants rested supine for less than 3 min before a 3-minute supine recording; acquisition followed the display of a usable preview signal. After rising, the 3-minute standing recording began within 1 min, and participants generally stood still. Participants breathed comfortably and spontaneously; respiratory rate and tidal volume were neither paced nor recorded. Center staff prohibited and controlled caffeine intake and physical exercise before testing. Medication information was obtained from guardian-provided records; medication was not discontinued for the study. The short stabilization and recording periods were selected for feasible assessment in this population and were not validated as equivalent to a conventional 5-minute resting protocol.

The manufacturer’s default signal-processing settings were used. Medicore stated that its processing follows the 1996 Task Force standards [18], including removal of detected ectopic beats and linear interpolation of affected beat intervals. The manufacturer described an exclusion threshold of three or more ectopic beats in a 3-minute recording. The conventional HF band is 0.15–0.40 Hz, and conventional normalization is HF (nu) = 100 × HF/(total power − very-low-frequency power (VLF)) [18,19]. The archived reports do not expose the exact band configuration, spectral algorithm, or acquisition sampling rate; these device-specific settings are therefore not asserted to have been independently verified.

ΔHR was standing minus supine mean heart rate; standing RMSSD and SDNN were taken from time-domain reports. HF (nu) was the device’s standing HF Norm output, not raw power in ms^2^ or logarithmic HF. Where LF/HF was available, HF Norm was checked against 100/(1 + LF/HF). Calculation failures, error codes (−1 or −1000), and nonphysiological zero values were treated as missing. Review flags were considered separately for each variable. One source-verified HF Norm output was retained while implausible heart-rate and time-domain outputs from the same recording were excluded from their corresponding analyses.

### 2.4. Gait Assessment

Gait was assessed with the F-Scan in-shoe plantar-pressure system (Tekscan, Inc., Norwood, MA, USA) at a sampling frequency of 100 HZ during three 6-meter straight walks at a comfortable self-selected speed [20]. Completion time was recorded separately for each trial, and trial-specific gait speed was calculated as 6 m divided by the corresponding completion time. Body weight was entered for each participant, and each foot was calibrated separately by lifting the opposite foot. Recording proceeded after successful calibration was confirmed. A walking trial was considered valid when the participant completed the entire 6-meter walkway at a self-selected speed, remained within the walkway, and plantar-pressure data were recorded throughout the walk. Trials were excluded if the pressure sensor failed to register data during the walk or if the participant left the walkway before completing the 6-meter distance. Trial-specific walking speeds are reported in Table A3. The three trial-specific speeds were first averaged for each participant, and the mean and sample standard deviation of these participant-level means were then calculated across the 21 participants.

Gait data were analyzed using the F-Scan software (Tekscan, Inc., Norwood, MA, USA, version 7.50). Original trials 1, 2, and 3 were exported from the Gait Parameters and Foot Parameters tables of F-Scan Analysis. The first and last detected stances were excluded from the averaging-range selection. This selection does not establish a measured 4-meter steady-state window or complete removal of all acceleration and deceleration effects. The software’s archived aggregate exports do not identify its event-detection threshold, so a particular force-threshold algorithm or visual event-by-event verification is not asserted. One analyzed participant received direct physical assistance during walking.

#### 2.4.1. Temporal Gait Variables, Variability, and Symmetry

Stride time was defined as the interval from initial contact of one foot to the subsequent initial contact of the same foot. Stance time was defined as the interval from initial contact to the last contact of the same foot, according to the F-Scan software definitions.

For each temporal gait variable, the F-Scan software provided a left-side mean (*L_t_*) and a right-side mean (*R_t_*) for each of the three 6-meter walking trials (t = 1, 2, 3). Calculation of the trial-to-trial coefficient of variation (CV) required all three trial-level bilateral means. The left- and right-side means were first averaged within each trial:

$$M_{t}\;=\;\frac{L_{t}\;+\;R_{t}}{2}$$

The mean and sample standard deviation of the three bilateral trial means were calculated as:

$$\bar{M}\;=\;\frac{M_{1}\;+\;M_{2}\;+\;M_{3}}{3}$$

$$s_{M}=\sqrt{\frac{{\left(M_{1}-\bar{M}\right)}^{2}+{\left(M_{2}-\bar{M}\right)}^{2}+{\left(M_{3}-\bar{M}\right)}^{2}}{3-1}}$$

The trial-to-trial CV was then calculated as

$$CV\;(\%)=100\times \frac{s_{M}}{\bar{M}}$$

Thus, this CV describes variation across the three trial-level bilateral means; it does not describe variation among consecutive strides within one trial.

To calculate the symmetry index (SI), the left- and right-side means were compared within each trial:

$${SI}_{t}\;(\%)\;=\;100\;\times \;\frac{\left|L_{t}\;-\;R_{t}\right|}{\frac{L_{t}\;+\;R_{t}}{2}}$$

The three trial-specific SIs were then averaged to obtain the participant-level SI:

$$SI\;(\%)\;=\;\frac{{SI}_{1}\;+\;{SI}_{2}\;+\;{SI}_{3}}{3}$$

An SI of 0% indicates that the left- and right-side means were equal within that trial. This SI summarizes left–right differences between trial-level means; it is not a cycle-matched comparison of individual strides or a measure of between-trial variability in SI.

Same-foot initial-contact-to-initial-contact (IC-to-IC) interval counts were checked retrospectively from the F-Scan display for the three analyzed trials. These counts refer to the software-detected contact sequence associated with the selected trial summaries, including the preceding IC when it was used to reproduce a selected stride-time output. Table A3 reports left- and right-side counts separately for each trial and their bilateral totals. Across 21 participants and 63 trials, 814 same-foot IC-to-IC intervals were counted (21–109 per participant across three trials). Three exported trial summaries were retained per participant, and these counts were not used to recalculate or weight the means, CVs, or SIs. The participant identified as G13 had 3, 3, and 15 bilateral intervals in trials 1, 2, and 3, respectively.

#### 2.4.2. Plantar Center-of-Force Trajectory Measures

The manufacturer defines COF deviation relative to the maximum medial-lateral distance of the center of force (COF) from the Foot Axis. FA (Foot Axis) is an axis running from the posterior Mid-Heel through the center of Metatarsal 2. Measurement is along the rows of the sensor. Lateral values are positive, medial values are negative. The F-Scan 3-Box output provided the maximum and minimum values. In this study, COF deviation was calculated by subtracting the minimum value from the maximum value; no absolute-value transformation was applied [21]. The resulting value is a derived mediolateral range between the exported endpoints and should be distinguished from the manufacturer-defined single maximum distance from the Foot Axis.

**Figure A1:**
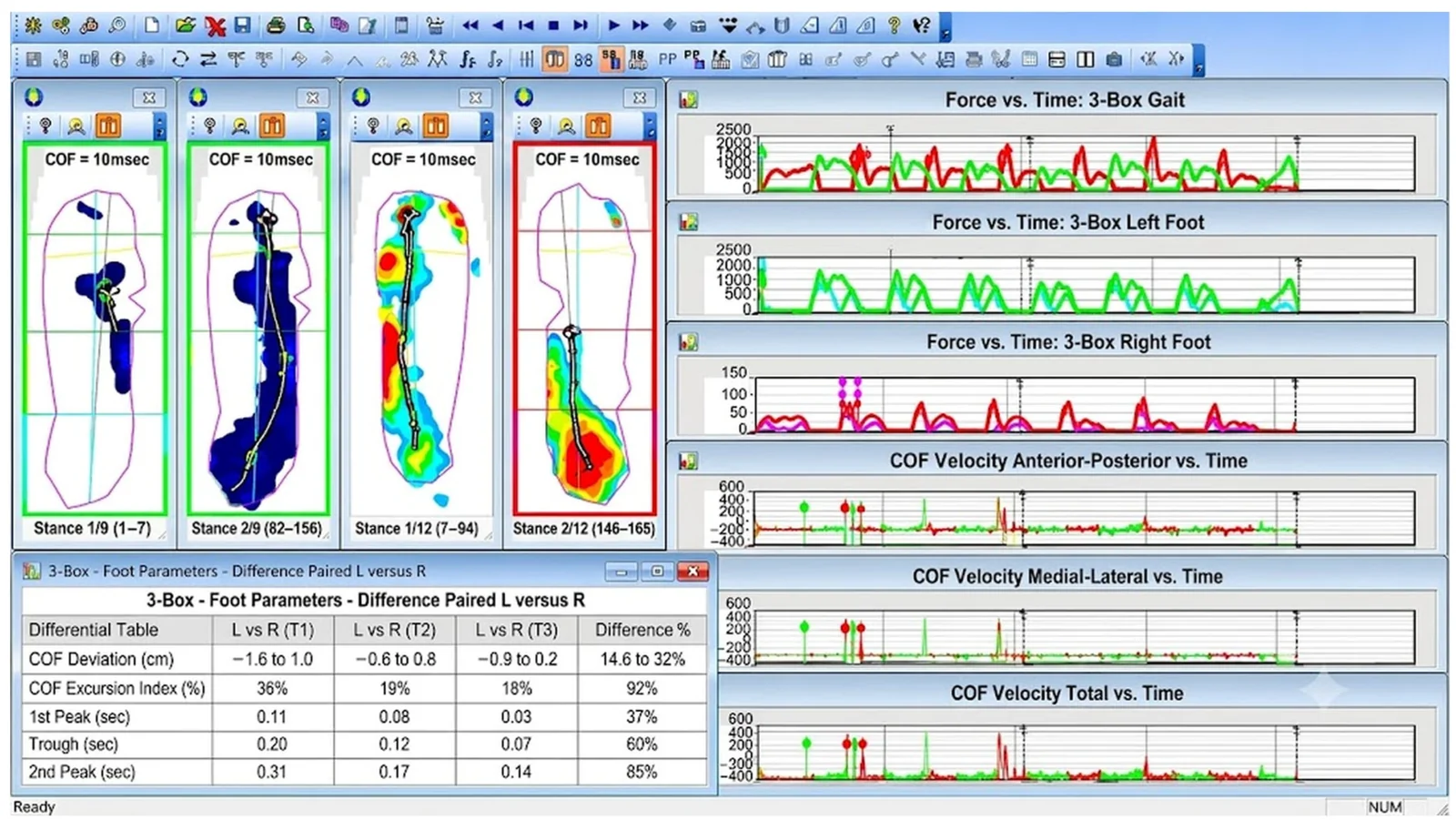
Anonymized F-Scan 3-Box Foot Parameters output showing COF Excursion Index percentages and signed COF deviation intervals. The first and second numerical columns show the left- and right-foot outputs, respectively. COF Excursion Index values are displayed as 36% and 19%; the corresponding COF deviation intervals are −1.6 to 1.0 cm and −0.6 to 0.8 cm. Identifying filenames are obscured, and numerical values are unchanged.

The manufacturer describes the COF Excursion Index as a positive percentage describing the concavity or mediolateral deviation of the center of force trajectory relative to foot width. The available manufacturer documentation defines the index conceptually but does not identify the exact numerator distance or provide the complete computational formula used by the software. The manual separately identifies WM as the width across the metatarsal heads or metatarsus; however, the available documentation does not explicitly state that this WM measure is the denominator used internally to calculate the COF Excursion Index. Therefore, the numerator and an unreported formula were not reconstructed, and WM was not assumed to be the denominator without manufacturer documentation. For each COF variable, left and right trial outputs were averaged first, and the three bilateral trial means were then averaged. For the COF Excursion Index, percentage values displayed by the F-Scan software were entered into the analysis dataset in decimal form (e.g., 36% as 0.36), and descriptive values are reported here on the percentage scale. An illustrative F-Scan 3-Box output is presented in Figure A1 to show the COF Excursion Index percentage values and the signed COF deviation intervals. These outputs describe plantar force trajectories rather than whole-body spatial stability.

### 2.5. Statistical Analysis

The primary analysis comprised 32 partial Spearman correlations between four cardiovascular–autonomic variables and eight gait variables, adjusted for age, sex, and body mass index (BMI). Age, sex, and BMI were included as common covariates to account for potential demographic and body-size influences on autonomic and gait measures; the small effective sample limits the precision of the adjusted estimates.

Correlations were calculated between covariate-adjusted residuals of ranked variables. Pairwise complete cases were used, yielding effective sample sizes of 16–18. Two-sided unadjusted *p* values were obtained using the t approximation with n − k − 2 degrees of freedom, where k denotes the number of covariates. Approximate 95% confidence intervals were calculated using Fisher’s z transformation as tanh [atan h(ρ) ± 1.96/√ (n − k − 3)]. These intervals are small-sample approximations, not bootstrap or multiplicity-adjusted intervals. The 32 primary correlations were treated as one family for BH-FDR adjustment, with adjusted *p* < 0.05 defining statistical significance. Both unadjusted and BH-FDR-adjusted *p* values are presented in Table A1.

ASD–ID comparisons were secondary and exploratory, using Mann–Whitney U tests for continuous variables and Fisher’s exact test for sex, without multiplicity adjustment.

Sensitivity analyses of the standing HF (nu)–stride time symmetry index association included additional adjustment for diagnosis and leave-one-participant-out analyses with ranks recalculated after each omission. Participant-level adjusted-rank residuals were plotted and included and excluded participant characteristics were summarized descriptively. Sensitivity analyses assessed individual influence and did not establish significance after multiple-testing correction. Statistical analyses were performed using IBM SPSS Statistics (version 30.0; IBM Corp., Armonk, NY, USA), and Benjamini–Hochberg false discovery rate (BH-FDR) adjustment was performed using Python (version 3.14.7; Python Software Foundation).

## 3. Results

### 3.1. Participants and Data Availability

Twenty-four adults enrolled (ASD *n* = 14; ID *n* = 10); mean age was 29.42 ± 4.99 years, 20 were men and four were women, and mean BMI was 26.88 ± 8.63 kg/m^2^. General assessment records were available for all 24. In week 1, gait testing was performed in 22; gait-test noncompletion affected two ID participants, because of absence (*n* = 1) or refusal (*n* = 1). One additional assessed ID participant, who used a walking aid, was excluded because of a technical measurement failure, leaving 21 available 3-Box datasets (ASD 14/14; ID 7/10).

Week 2 HRV source records covered 24 participants. Three calculation failures left 21 HF Norm outputs; one additional implausible time-domain record left 20 RMSSD/SDNN cases, and three additional heart-rate QC exclusions left 18 ΔHR cases.

After pairing with gait data, the correlation samples were 18 for HF (nu), 17 for RMSSD/SDNN, and 16 for ΔHR. These are intersections, not sequential exclusions. Figure 1 and Table 1 and Table 2 summarize availability.

Descriptive characteristics of participants with and without usable data for each autonomic-variable analysis are presented in Table A4. For the standing HF (nu)–stride time SI subset, the 18 included participants comprised 11 with ASD and seven with ID, and 15 men and three women; the six excluded participants comprised three with ASD and three with ID, and five men and one woman. Mean age was 29.94 ± 5.51 versus 27.83 ± 2.71 years, and BMI was 27.11 ± 8.28 versus 26.17 ± 10.40 kg/m^2^. These descriptive comparisons do not establish equivalence or exclude selection bias.

### 3.2. Partial Correlations Between Upright Cardiovascular-Autonomic Characteristics and Gait Parameters

**Figure 2:**
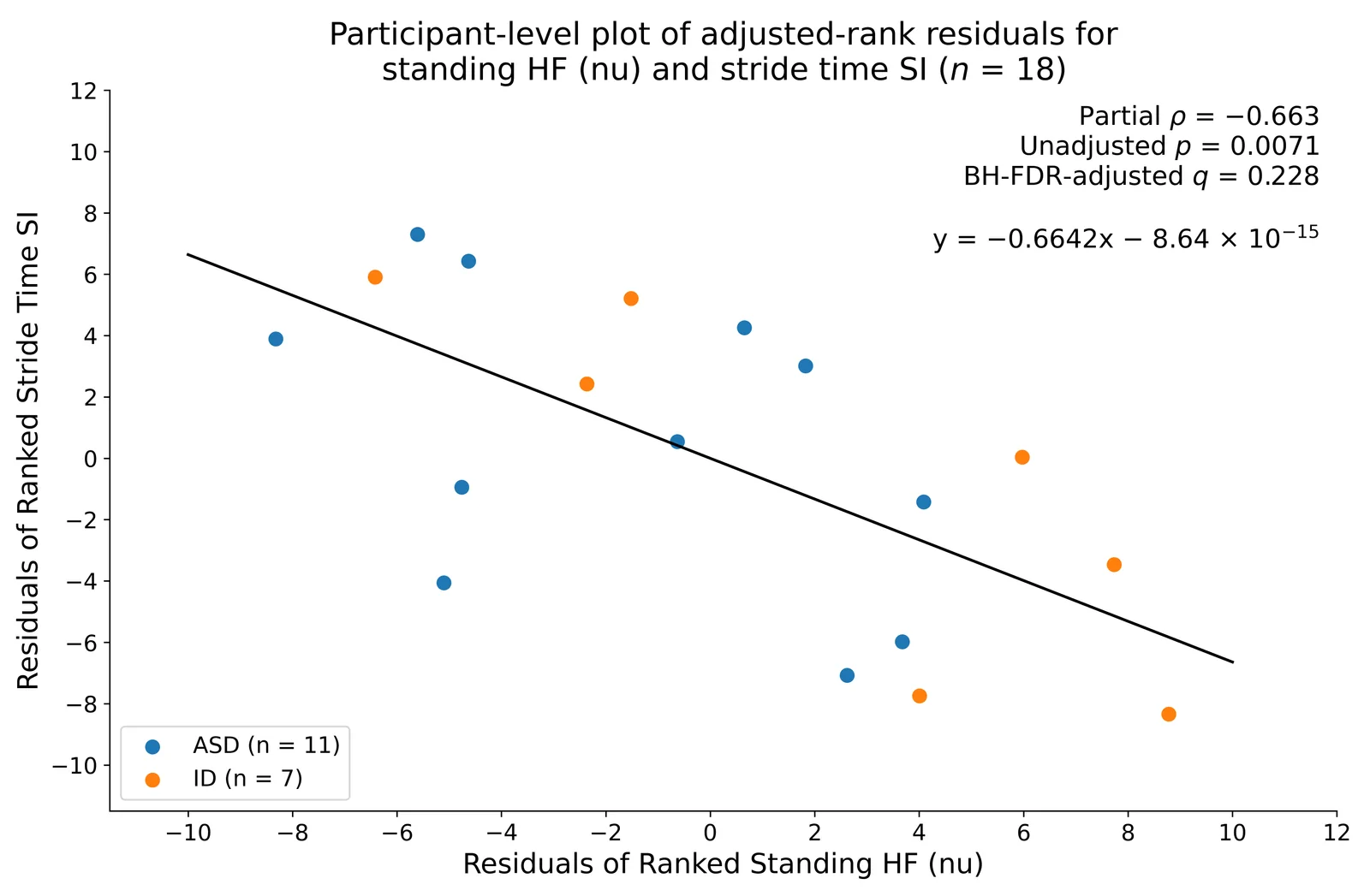
Participant-level plot of adjusted-rank residuals for standing HF (nu) and stride time SI (*n* = 18). Each point represents one participant, and both axes show residuals of ranked variables adjusted for age, sex, and BMI. Colors indicate diagnostic groups (ASD, *n* = 11; ID, *n* = 7) for descriptive visualization only. The fitted line illustrates the observed relationship for the overall sample descriptively (partial ρ = −0.663, unadjusted *p* = 0.0071; BH-FDR-adjusted q = 0.228). Diagnostic-group coloring does not represent separate group-specific regression analyses.

Among 32 age-, sex-, and BMI-adjusted correlations, standing HF (nu) and stride time SI showed a nominal negative association (ρ = −0.663, unadjusted *p* = 0.0071, *n* = 18; approximate 95% CI −0.877 to −0.227). It did not survive BH-FDR correction (q = 0.228), and no primary pair reached q < 0.05. Additional diagnosis adjustment yielded ρ = −0.672 (unadjusted *p* = 0.0085, *n* = 18, df = 12; approximate 95% CI −0.886 to −0.220). Leave-one-participant-out coefficients ranged from −0.767 to −0.559, with all nominal *p* values below 0.05 (Table A2 and Figure 2).

The participant who received direct physical assistance during gait assessment was included in the primary analysis. After excluding this participant, the association between standing HF (nu) and stride time SI remained negative (partial ρ = −0.626, unadjusted *p* = 0.0167, *n* = 17). These checks describe individual influence but do not prove robustness to measurement error or corrected significance. Excluding the reviewed standing HF Norm observation yielded partial ρ = −0.626 (unadjusted *p* = 0.0167, *n* = 17). Excluding the participant with sparse IC-to-IC intervals in the first two gait trials yielded partial ρ = −0.614 (unadjusted *p* = 0.0196, *n* = 17). These checks are two identified omissions within Table A2 and do not establish significance after multiple-testing correction.

Standing RMSSD and stance time CV were not significantly associated (ρ = −0.527, unadjusted *p* = 0.053, *n* = 17). No nominally significant associations were observed for ΔHR or the COF measures (Table 3). All 32 coefficients, exact nominal *p* values, approximate 95% confidence intervals, and BH-FDR q values are provided in Table A1; non-significant results do not establish absence of an association.

### 3.3. Exploratory Comparison Between Diagnostic Groups

Of the 24 participants, 14 had ASD and 10 had ID (Table 4). Age and BMI did not differ significantly between diagnostic groups, whereas the sex distribution differed (ASD male/female, 14/0; ID, 6/4; Fisher’s exact *p* = 0.020). None of the primary autonomic or gait variables differed between groups at *p* < 0.05.

Standing HF (nu) was numerically lower in the ASD group (11.22 [7.15–22.83] nu) than in the ID group (35.39 [15.27–44.42] nu), but this difference did not reach the conventional significance threshold (U = 17.0, *p* = 0.057).

No significant group differences were found for COF deviation or excursion, or for the mean, CV, or SI of stride and stance time (all *p* > 0.05).

These unadjusted group comparisons were secondary and exploratory. None of the enrolled participants had coexisting ASD and ID diagnoses; no allocation rule for participants with both diagnoses was required. Diagnosis-related and sex-related differences cannot be reliably separated because all ASD participants were male; non-significance does not establish similarity or equivalence.

## 4. Discussion

This exploratory study examined upright cardiovascular-autonomic characteristics alongside temporal gait measures and plantar COF measures in adults with ID or ASD. Standing HF (nu) and stride time SI showed a nominal negative association, with a similar direction after diagnosis adjustment. However, no primary association survived correction across 32 tests. The HF (nu)–SI estimate therefore remains an exploratory hypothesis; its nominal significance does not establish a stronger temporal than spatial relationship.

A significant nominal result for one variable pair and non-significant results for other pairs do not constitute a statistical comparison of domains. CPG activity, vestibular cortical activity, and cardiac–locomotor phase synchronization were not measured, so these mechanisms remain background hypotheses rather than explanations established by the data. The early standing acquisition, short HRV recording, and unmonitored spontaneous respiration further constrain physiological interpretation.

Walking automaticity and attentional demands provide context for future research [22]. Dual-task gait differences in ID [10] suggest that temporal measures may depend on task conditions. Cognitive functioning, dual-task performance, and everyday mobility were not measured here, so the present association cannot establish a functional benefit or reduced attentional demand. A meta-analysis links social interaction conditions with HRV [23], but social stress and emotional state were not measured in this study. Their possible influence is a hypothesis for future work and cannot explain the observed association from these data. Standing RMSSD and stance time CV were not significantly associated, providing insufficient evidence of an association in this sample.

Temporal CV describes variation among three bilateral trial summaries rather than stride-to-stride variation. SI averages three bilateral trial-summary differences without matching individual left and right cycles. Neither trial averaging nor a low SI independently establishes reliability or functional stability. Longer event-level recordings with explicit cycle alignment are needed to assess these distinctions.

Previous research in healthy adults showed that slower walking speeds were associated with greater stride-to-stride time variability [24]. This finding provides context for reporting walking speed alongside temporal gait measures, although it does not directly validate the trial-to-trial CV used here. A study using foot-worn inertial sensors in healthy adults also found that reliability differed between spatiotemporal measures and symmetry indices [25]; these results cannot establish the reliability of the F-Scan summaries in the present population.

ΔHR was not significantly associated with any gait measure. As a difference between supine and standing mean heart rate, it does not directly measure sympathetic activity, baroreflex sensitivity, stroke volume, or venous return. Non-significance does not establish irrelevance to gait. Prior studies of orthostatic responses in ID [16], autonomic–postural regulation in older adults [26], and heart-rate behavior during walking [27] motivate further research but do not show that one autonomic measure is more closely related to gait than another in this sample.

COF deviation and COF excursion were not significantly associated with any of the four cardiovascular–autonomic variables. Temporal gait summaries and plantar COF measures describe different measured characteristics. Previous research has identified contributions of ankle muscles to mediolateral center-of-pressure control during walking [11], but the present study did not assess these mechanisms.

Moreover, the COF measures derived from the F-Scan 3-Box Analysis output are not equivalent to whole-body center of mass or force-platform center of pressure. The present findings therefore should not be generalized to imply that spatial stability is unrelated to autonomic regulation. Future studies should combine plantar COF with force-platform CoP, whole-body kinematics, and controlled sensory conditions to evaluate temporal and spatial gait regulation and autonomic responses more comprehensively.

Diagnostic-group comparisons were secondary, unadjusted, and imprecise. Prior work describes gait and perturbation-response differences in ID [9,10,28] and motor, sensory, and postural differences in ASD [6,29,30,31]. Our nonsignificant tests neither contradict these findings nor establish that the groups are similar. The small samples, heterogeneous participants, and sex imbalance limit diagnosis-specific interpretation.

The joint assessment can inform future research questions, but laboratory CV, SI, and COF descriptors are distinct from falls, balance, dual-task performance, and community mobility. These outcomes and treatment responses were not assessed. The present exploratory data therefore do not support clinical assessment recommendations or an autonomic or breathing intervention; such applications require independent prospective evaluation.

All correlations were analyzed within the same study cohort. Differences in effective sample size (n = 16–18) arose because device calculation failures or invalid outputs made some variable-specific measurements unavailable, rather than because participants were selected separately for each correlation.

None of the 32 associations survived BH-FDR correction. Diagnostic groups differed in sex composition, and all three unavailable gait datasets occurred in the ID group, leaving gait data available for 7/10 ID participants and 14/14 ASD participants. This differential availability may underrepresent ID participants who could not complete gait assessment and may introduce selection bias into the correlations and diagnostic-group comparisons. The direction and magnitude of this potential bias cannot be determined from the available data.

Cognitive functioning was not assessed, and the cross-sectional design precludes causal inference. Three-minute HRV recordings after short supine stabilization and within 1 min of standing, together with unmonitored respiratory rate and tidal volume, introduce uncertainty in HF (nu) interpretation.

Medication use was recorded, while individual medication effects, epilepsy, intellectual functioning, and habitual physical activity were not fully controlled. The reviewed HF Norm output was source-verified but not independently validated physiologically. Unequal software-detected IC-to-IC interval counts, physical assistance in one participant, and sparse early-trial IC-to-IC intervals in another constrain stride SI interpretation. Future work should retain beat-level and gait-event quality logs, use longer recordings, and directly assess cognitive functioning, balance, and real-world mobility.

## 5. Conclusions

This exploratory study jointly assessed upright cardiovascular–autonomic characteristics and gait measures within the same cohort of adults with ID or ASD, providing an integrated perspective on these domains. Standing HF (nu) showed a nominal negative association with stride time SI, but none of the 32 primary correlations remained significant after BH-FDR correction. Given the small effective sample and sex imbalance, this observation remains hypothesis-generating and provides a basis for further investigation of autonomic characteristics and bilateral gait timing in adults with developmental disabilities. The findings do not establish stronger associations with temporal than plantar COF measures or demonstrate functional stability, fall risk, or daily mobility outcomes. Larger studies with comprehensive HRV acquisition, respiratory monitoring, validated gait-event measurements, and direct cognitive and balance assessments are needed.

## Figures and Tables

**Table 1:** General characteristics of the participants.

Variable	*n*	Value
Age (years)	24	29.42 ± 4.99
Sex (male/female)	24	20/4
Diagnosis (ID/ASD)	24	10/14
BMI (kg/m^2^)	24	26.88 ± 8.63

Continuous variables are presented as mean ± standard deviation. Sample size (*n*) represents the number of valid cases available for each variable. ASD, autism spectrum disorder; BMI, body mass index; ID, intellectual disability.

**Table 2:** Descriptive statistics for the primary analysis variables.

Variable	*n*	Value
ΔHR (bpm)	16	11.56 ± 8.16
Standing RMSSD (ms)	17	32.02 ± 24.67
Standing SDNN (ms)	17	43.01 ± 19.62
Standing HF (nu)	18	21.14 ± 15.88
Walking Speed (m/s)	21	0.830 ± 0.189
COF Deviation (cm)	21	1.70 ± 0.68
COF Excursion Index (%)	21	21.9 ± 7.7
Stride Time Mean (s)	21	1.209 ± 0.299
Stride Time CV (%)	21	10.96 ± 11.52
Stride Time SI (%)	21	11.00 ± 8.62
Stance Time Mean (s)	21	0.728 ± 0.271
Stance Time CV (%)	21	10.37 ± 10.42
Stance Time SI (%)	21	17.17 ± 20.33

Continuous variables are presented as mean ± standard deviation. Sample size (*n*) represents the number of valid cases available for each variable. ΔHR, change in heart rate from supine to standing; COF, center of force; CV, coefficient of variation; HF, high frequency; RMSSD, root mean square of successive differences; SDNN, standard deviation of normal-to-normal intervals; SI, symmetry index. HRV sample sizes in this table refer to the paired gait-analysis samples (16–18), whereas standalone usable HRV counts before pairing were 18–21 (Figure 1).

**Table 3:** Age-, sex- and BMI-adjusted partial Spearman rank correlations between cardiovascular-autonomic and gait variables.

Variable	COF Dev.	COF Exc.	Stride Mean	Stride CV	Stride SI	Stance Mean	Stance CV	Stance SI
ΔHR	0.421 (0.152)	0.295 (0.328)	0.001 (0.997)	−0.227 (0.455)	0.086 (0.780)	0.021 (0.947)	0.106 (0.731)	0.396 (0.180)
Standing RMSSD	−0.057 (0.846)	−0.044 (0.882)	−0.067 (0.820)	−0.344 (0.228)	−0.311 (0.280)	−0.264 (0.362)	−0.527 (0.053)	−0.081 (0.782)
Standing SDNN	−0.098 (0.740)	−0.090 (0.760)	−0.005 (0.986)	−0.376 (0.185)	−0.162 (0.579)	−0.080 (0.784)	−0.475 (0.086)	−0.226 (0.437)
Standing HF (nu)	0.048 (0.864)	0.015 (0.957)	0.184 (0.512)	−0.089 (0.753)	−0.663 * (0.007)	0.100 (0.724)	−0.452 (0.091)	−0.461 (0.084)

Each cell presents the partial Spearman coefficient and the nominal two-sided *p* value in parentheses. The original 32-test coefficients are retained; BH-FDR-adjusted q values are provided in Table A1. RMSSD, root mean square of successive differences; SDNN, standard deviation of normal-to-normal intervals; HF, high frequency; COF, center of force; CV, coefficient of variation; Dev., deviation; Exc., excursion; SI, symmetry index. Effective sample sizes were 16 for ΔHR, 17 for RMSSD/SDNN, and 18 for HF (nu). * Nominal *p* < 0.05; no primary association reached BH-FDR q < 0.05.

**Table 4:** Exploratory comparison between diagnostic groups.

Variable	ASD		ID		*p* Value
	N	Median	N	Median	
Age (years)	14	28.5 [26.0–31.8]	10	28.0 [26.2–30.2]	0.616
Sex (male/female)	14	14/0	10	6/4	0.020
BMI (kg/m^2^)	14	27.15 [22.90–31.35]	10	21.22 [18.47–31.60]	0.143
ΔHR (bpm)	10	12.50 [5.00–15.50]	6	13.00 [10.75–16.00]	0.703
Standing RMSSD (ms)	11	17.99 [13.06–36.53]	6	36.38 [16.81–64.83]	0.514
Standing SDNN (ms)	11	36.25 [24.16–46.37]	6	64.16 [41.11–65.62]	0.248
Standing HF (nu)	11	11.22 [7.15–22.83]	7	35.39 [15.27–44.42]	0.057
COF Deviation (cm)	14	1.61 [1.22–2.08]	7	1.60 [0.94–2.27]	0.654
COF Excursion Index (%)	14	20.2 [16.8–26.8]	7	23.0 [13.5–27.4]	0.794
Stride Time Mean (s)	14	1.192 [1.088–1.277]	7	1.153 [1.062–1.224]	0.433
Stride Time CV (%)	14	8.36 [4.37–11.34]	7	9.91 [4.77–11.63]	0.970
Stride Time SI (%)	14	7.73 [5.11–10.96]	7	8.58 [5.80–19.33]	0.526
Stance Time Mean (s)	14	0.709 [0.623–0.795]	7	0.632 [0.577–0.676]	0.168
Stance Time CV (%)	14	8.90 [3.44–12.44]	7	5.15 [4.13–12.28]	0.682
Stance Time SI (%)	14	8.06 [3.43–19.97]	7	15.98 [7.26–22.59]	0.391

Continuous variables are presented as N, median [interquartile range] and were compared using the Mann–Whitney U test. Sex is presented as male/female frequency and was compared using Fisher’s exact test. Sample sizes reflect the number of valid observations available for each variable. No multiplicity correction was applied to these secondary diagnostic-group comparisons; BH-FDR correction of the 32 primary correlations is reported in Table A1. ASD, autism spectrum disorder; BMI, body mass index; COF, center of force; CV, coefficient of variation; HF, high frequency; ID, intellectual disability; RMSSD, root mean square of successive differences; SDNN, standard deviation of normal-to-normal intervals; SI, symmetry index; ΔHR, change in heart rate from supine to standing.

## Data Availability

The data presented in this study are available from the corresponding author upon reasonable request. The data are not publicly available because they include sensitive health information from a vulnerable participant population and are subject to the approved ethical procedures and applicable privacy requirements.

## Abbreviations

ASD	autism spectrum disorder
BMI	body mass index
COF	center of force
CV	coefficient of variation
HF (nu)	normalized high-frequency component
HR	heart rate
HRV	heart-rate variability
ID	intellectual disability
RMSSD	root mean square of successive differences
SDNN	standard deviation of normal-to-normal intervals
SI	symmetry index
ΔHR	standing heart rate minus supine heart rate

## Appendix A

### Appendix A.1

**Table A1:** All 32 primary partial correlations with approximate 95% confidence intervals and BH-FDR correction.

Autonomic Variable	Gait Variable	*n*	Partial ρ	Nominal *p*	BH-FDR q	Approx. 95% CI
ΔHR	COF deviation	16	0.421	0.151799	0.739383	−0.169 to 0.789
ΔHR	COF excursion	16	0.295	0.328004	0.954194	−0.306 to 0.728
ΔHR	Stride mean	16	0.001	0.996890	0.996890	−0.550 to 0.552
ΔHR	Stride CV	16	−0.227	0.454885	0.996890	−0.692 to 0.370
ΔHR	Stride SI	16	0.086	0.779575	0.996890	−0.488 to 0.608
ΔHR	Stance mean	16	0.021	0.946615	0.996890	−0.536 to 0.565
ΔHR	Stance CV	16	0.106	0.731058	0.996890	−0.473 to 0.621
ΔHR	Stance SI	16	0.396	0.180387	0.739383	−0.198 to 0.777
Standing RMSSD	COF deviation	17	−0.057	0.845747	0.996890	−0.571 to 0.488
Standing RMSSD	COF excursion	17	−0.044	0.882485	0.996890	−0.561 to 0.499
Standing RMSSD	Stride mean	17	−0.067	0.820258	0.996890	−0.577 to 0.481
Standing RMSSD	Stride CV	17	−0.344	0.228139	0.811163	−0.740 to 0.228
Standing RMSSD	Stride SI	17	−0.311	0.279865	0.895567	−0.722 to 0.263
Standing RMSSD	Stance mean	17	−0.264	0.362221	0.965924	−0.697 to 0.310
Standing RMSSD	Stance CV	17	−0.527	0.052574	0.580217	−0.827 to 0.004
Standing RMSSD	Stance SI	17	−0.081	0.782397	0.996890	−0.587 to 0.470
Standing SDNN	COF deviation	17	−0.098	0.739809	0.996890	−0.597 to 0.457
Standing SDNN	COF excursion	17	−0.090	0.760187	0.996890	−0.592 to 0.463
Standing SDNN	Stride mean	17	−0.005	0.985738	0.996890	−0.534 to 0.527
Standing SDNN	Stride CV	17	−0.376	0.184846	0.739383	−0.756 to 0.193
Standing SDNN	Stride SI	17	−0.162	0.579287	0.996890	−0.638 to 0.403
Standing SDNN	Stance mean	17	−0.080	0.784430	0.996890	−0.586 to 0.470
Standing SDNN	Stance CV	17	−0.475	0.086006	0.580217	−0.803 to 0.074
Standing SDNN	Stance SI	17	−0.226	0.437416	0.996890	−0.676 to 0.346
Standing HF (nu)	COF deviation	18	0.048	0.864488	0.996890	−0.476 to 0.547
Standing HF (nu)	COF excursion	18	0.015	0.956986	0.996890	−0.501 to 0.523
Standing HF (nu)	Stride mean	18	0.184	0.511917	0.996890	−0.363 to 0.636
Standing HF (nu)	Stride CV	18	−0.089	0.753199	0.996890	−0.575 to 0.444
Standing HF (nu)	Stride SI	18	−0.663	0.007115	0.227690	−0.877 to −0.227
Standing HF (nu)	Stance mean	18	0.100	0.723626	0.996890	−0.435 to 0.582
Standing HF (nu)	Stance CV	18	−0.452	0.090659	0.580217	−0.783 to 0.078
Standing HF (nu)	Stance SI	18	−0.461	0.083990	0.580217	−0.787 to 0.068

All rows belong to one 32-test family; no q value was below 0.05. Confidence intervals are unadjusted Fisher-transform approximations for the partial rank coefficients and do not establish familywise or FDR-adjusted confidence coverage. Abbreviations: ΔHR, change in heart rate from supine to standing; RMSSD, root mean square of successive differences; SDNN, standard deviation of normal-to-normal intervals; HF, high frequency; COF, center of force; CV, coefficient of variation; SI, symmetry index.

### Appendix A.2

**Table A2:** Leave-one-participant-out sensitivity analysis.

Omitted ID	*n*	Partial ρ	Nominal *p*
P01	17	−0.647620	0.012274
P02	17	−0.648271	0.012158
P03	17	−0.651803	0.011545
P04	17	−0.668076	0.009017
P05	17	−0.650834	0.011711
P06	17	−0.675229	0.008053
P07	17	−0.653233	0.011303
P08	17	−0.625758	0.016680
P09	17	−0.558992	0.037703
P10	17	−0.705532	0.004816
P11	17	−0.670213	0.008720
P12	17	−0.684969	0.006869
P13	17	−0.613590	0.019603
P14	17	−0.766527	0.001384
P15	17	−0.633139	0.015077
P16	17	−0.704740	0.004885
P17	17	−0.649508	0.011941
P18	17	−0.630189	0.015703

P01–P18 are anonymized row identifiers for this pair, not enrollment IDs. Ranks and covariate residuals were recalculated in every subset. Nominal *p* values do not replace the primary BH-FDR results. P08 is the omission of the reviewed standing HF Norm observation; P13 is the omission of the participant with sparse IC-to-IC intervals in the first two gait trials. These *p* identifiers refer to the plot/leave-one-out sample and are distinct from the G identifiers in Table A3.

### Appendix A.3

**Table A3:** Same-foot IC-to-IC interval counts in the three analyzed F-Scan trials.

Gait ID	Trial 1	Speed (m/s)	Trial 2	Speed (m/s)	Trial 3	Speed (m/s)	Total
G01	7/10 (17)	0.880	7/7 (14)	0.756	6/7 (13)	0.845	44
G02	6/5 (11)	0.739	6/6 (12)	0.888	5/4 (9)	1.068	32
G03	4/5 (9)	0.917	4/5 (9)	0.946	5/4 (9)	0.908	27
G04	5/6 (11)	0.744	6/6 (12)	0.715	6/6 (12)	0.651	35
G05	4/4 (8)	0.877	5/6 (11)	0.816	4/5 (9)	1.010	28
G06	4/5 (9)	1.195	4/4 (8)	1.054	5/4 (9)	1.014	26
G07	7/7 (14)	0.812	6/6 (12)	0.868	6/5 (11)	0.901	37
G08	14/20 (34)	0.477	15/19 (34)	0.575	18/23 (41)	0.569	109
G09	7/7 (14)	0.787	8/8 (16)	0.669	8/8 (16)	0.700	46
G10	7/9 (16)	0.524	8/8 (16)	0.540	8/8 (16)	0.589	48
G11	5/5 (10)	0.809	5/6 (11)	0.780	6/6 (12)	0.793	33
G12	10/13 (23)	0.361	9/10 (19)	0.477	8/9 (17)	0.515	59
G13	1/2 (3)	0.494	1/2 (3)	0.613	8/7 (15)	0.566	21
G14	8/6 (14)	0.768	5/5 (10)	0.898	4/5 (9)	0.892	33
G15	5/5 (10)	1.266	7/6 (13)	0.949	8/5 (13)	0.958	36
G16	6/7 (13)	0.831	8/8 (16)	0.842	7/7 (14)	0.837	43
G17	4/3 (7)	0.915	4/4 (8)	0.920	5/5 (10)	1.193	25
G18	7/7 (14)	0.806	6/5 (11)	1.143	5/5 (10)	1.212	35
G19	6/6 (12)	0.696	6/5 (11)	0.729	5/6 (11)	0.876	34
G20	6/7 (13)	1.071	4/4 (8)	1.399	6/7 (13)	0.738	34
G21	5/6 (11)	0.919	4/5 (9)	0.980	4/5 (9)	1.026	29
All participants	273	—	263	—	278	—	814

Notes: Each trial cell reports left/right IC-to-IC interval counts, followed by the bilateral total in parentheses. The final column reports the total across three trials. Counts were checked from the software-displayed contact sequence for the selected trial summaries; the preceding IC was included where it contributed to reproduction of a selected stride-time output. No interval connects separate trials. These counts describe the amount of software-detected gait observation and do not independently establish valid physiological gait cycles. Left and right intervals were counted separately and were not matched as bilateral cycle pairs. Three trial summaries per participant were retained without weighting by interval count. G13 had sparse early-trial intervals; its exported values were retained. G01–G21 are anonymized gait-data identifiers. Trial-specific walking speeds (m/s), calculated as 6 m divided by the corresponding completion time, are also reported for each of the three trials.

### Appendix A.4

**Table A4:** Descriptive characteristics of included and excluded participants.

Variable Subset	Status	*n*	Age (Years)	BMI (kg/m^2^)	Male/Female	ASD/ID
ΔHR	Included	16	29.94 ± 5.86	26.51 ± 8.02	14/2	10/6
ΔHR	Excluded	8	28.38 ± 2.56	27.61 ± 10.29	6/2	4/4
RMSSD/SDNN	Included	17	29.88 ± 5.68	27.34 ± 8.48	15/2	11/6
RMSSD/SDNN	Excluded	7	28.29 ± 2.75	25.75 ± 9.56	5/2	3/4
HF (nu)	Included	18	29.94 ± 5.51	27.11 ± 8.28	15/3	11/7
HF (nu)	Excluded	6	27.83 ± 2.71	26.17 ± 10.40	5/1	3/3

Included means contributing to the corresponding pairwise-complete primary correlations; excluded means not contributing among the 24 enrolled participants. Age and BMI are mean ± sample SD. RMSSD and SDNN used identical participant sets. These are descriptive comparisons without additional significance or equivalence tests. Abbreviations:ΔHR, change in heart rate from supine to standing; RMSSD, root mean square of successive differences; SDNN, standard deviation of normal-to-normal intervals; HF, high frequency.
